# Integrated transcriptomic analysis reveals miRNA-hub mRNA-TF interactions and key regulatory targets in *STEC* infected intestinal epithelial cells

**DOI:** 10.3389/fcimb.2026.1772607

**Published:** 2026-04-02

**Authors:** Baili Zheng, Xiaoyue Su, Yongchao Li, Qiang Fu, Xuelian Ma, Bao Zhou, Bing Peng, Rulong Chen, Yingyu Liu

**Affiliations:** 1 College of Veterinary Medicine, Xinjiang Key Laboratory of Herbivore Drug Research and Creation, Xinjiang Agricultural University, Urumqi, China; 2 Bayinguoleng Vocational and Technical College, Korla, Bayinguoleng, China; 3 Xinjiang Tianlai Agricultural and Animal Husbandry Group Co., Ltd., Bole, China

**Keywords:** gene regulatory networks, microRNA, regulatory target, RNA sequencing, Shiga toxin-producing *Escherichia coli*

## Abstract

**Background:**

Shiga toxin-producing *Escherichia coli* (*STEC*) is a leading foodborne pathogen responsible for hemolytic uremic syndrome (HUS). This pathogen poses a severe threat to global public health. MicroRNAs (miRNAs) are increasingly recognized as essential post-transcriptional regulators and therapeutic targets. Despite this, their exact regulatory networks and roles in STEC pathogenesis remain largely unknown.

**Methods:**

An *in vitro* infection model was established using human intestinal epithelial cells (HIECs), with optimal infection time and bacterial load determined via CCK-8 assays and DAPI staining. High-throughput RNA sequencing (RNA-seq) was performed to profile miRNA and mRNA expression, followed by RT-qPCR validation. Differentially expressed miRNAs and their target mRNAs were identified by integrating miRanda predictions with transcriptomic data. Target functions were annotated using GO and KEGG enrichment analyses. A protein–protein interaction (PPI) network was constructed to identify core hub genes, and upstream transcription factors (TFs) were predicted using the TRRUST and hTFtarget databases, culminating in the construction of an integrated miRNA–hub mRNA–TF regulatory network.

**Results:**

Infection of HIECs with STEC (10^6^ CFU/mL) for 1 hour induced profound cellular structural damage, accompanied by the differential expression of 652 target mRNAs (301 upregulated and 351 downregulated). Functional enrichment revealed that these targets are predominantly involved in inflammatory responses, apoptosis, and cell proliferation. Through the PPI network, 10 core hub genes (including TNF, CXCL8, CCN2, and TGFB2) were identified, along with 11 highly correlated regulatory TFs. Based on the integrated network analysis, has-miR-3121-3p, hsa-miR-219b-5p, and hsa-miR-543 were pinpointed as master regulatory miRNAs, suggesting they orchestrate critical host signaling pathways during infection.

**Conclusion:**

STEC infection drastically reprograms the transcriptomic landscape of HIECs, triggering the dysregulation of inflammation and apoptosis-related pathways. This study is the first to delineate a comprehensive miRNA–mRNA–TF regulatory network for STEC infection, highlighting miR-3121-3p, miR-219b-5p, and miR-543 as key molecular mediators. These findings provide novel insights into the molecular pathogenesis of STEC and lay a crucial foundation for exploring potential regulatory and host-directed therapeutic targets.

## 1 Introduction

Shiga toxin-producing *Escherichia coli* (*STEC*) is a zoonotic pathogen that causes foodborne illness in both humans and animals (Dias et al., 2022). It represents as the third most prevalent causative agent of global foodborne zoonotic diseases and is a primary cause of diarrhea-associated hemolytic uremic syndrome (HUS) in humans (Mathusa et al., 2010; Scallan et al., 2011; Liu et al., 2022). Cattle serve as the main natural reservoirs of *STEC*. Human infections arise from the consumption of food, water, or vegetables contaminated with animal feces, or through direct contact with infected animals (Gonzalez and Cerqueira, 2020). Worldwide, *STEC* infections cause an estimated 2.8 million acute cases annually, presenting a significant public health challenge (Onyeka et al., 2020). As a result, *STEC* has emerged as a global public health concern.

*STEC* produces characteristic attaching-and-effacing (AE) lesions on the surface of human intestinal epithelial cells (HIECs) (Alharbi et al., 2022). These lesions disrupt the integrity of the intestinal epithelial barrier, impairing its structural and functional properties, leading to cellular damage, and altering normal intestinal physiology (FAO/WHO STEC Expert Group, 2019). Although antibiotics are conventionally used in the treatment of bacterial infections, their clinical application in *STEC* management remains highly controversial. Beyond the escalating concern of antimicrobial resistance, experimental studies have shown that specific antibiotic classes, notably quinolones that disrupt bacterial DNA replication, may unintentionally induce increased Shiga toxin (Stx) production, consequently heightening the risk of severe disease manifestations (Zhang et al., 2000; Kennedy et al., 2017; Gelalcha et al., 2022). Therefore, it is essential to explore novel molecular-level strategies, with particular emphasis on identifying potential cellular regulatory targets associated with *STEC* infection. These key molecular hubs could serve as a theoretical basis and provide candidate molecules for precision therapy and targeted intervention.

MicroRNAs (miRNAs) are a class of endogenous, non-coding small RNAs that regulate target gene expression, primarily through negative regulation, and play essential roles in diverse biological processes such as cell proliferation, apoptosis, development, disease pathogenesis, and therapeutic responses (Cardoso et al., 2021). Intracellularly, miRNAs orchestrate intricate feedback regulatory networks. Owing to their robust expression profiles and high disease specificity, miRNAs have emerged not only as reliable diagnostic biomarkers for diverse pathologies but also as actionable therapeutic targets currently advancing through multiple clinical trials (Khamaneh et al., 2025; Batsaikhan et al., 2025). Key miRNAs function as key post transcriptional regulators, coordinating the expression of extensive downstream gene networks. Accumulating evidence indicates that during bacterial infection, Key miRNAs play a conserved and critical role in coordinating host immune responses and metabolic reprogramming (Yang et al., 2019). Nevertheless, the expression dynamics and regulatory roles of miRNAs in *STEC* infection remain poorly characterized. Identifying potential regulatory targets of miRNAs associated with *STEC* infection would provide important insights into its pathogenic mechanisms and furnish essential molecular underpinnings for host-directed therapies and targeted interventions.

In this study, RNA sequencing was employed to conduct transcriptomic profiling of HIECs following *STEC* infection. Integrated analysis revealed critical miRNAs exhibiting core regulatory functions during *STEC* infection, thereby establishing a theoretical foundation for their development as potential therapeutic targets.

## 2 Materials and methods

### 2.1 Research materials

The *STEC* CD15-H34 strain was maintained at −80^°^C in Luria Bertani (LB) broth supplemented with 30% glycerol at the College of Veterinary Medicine, Xinjiang Agricultural University. The HIECs were obtained from Shanghai Guandao Biological Engineering Co., Ltd. (Shanghai, China). Trypsin (0.25%) was purchased from Biological Industries (Beit HaEmek, Israel).

### 2.2 Cell culture and bacterial strains

HIEC cells were cultured in RPMI-1640 medium supplemented with 10% heat-inactivated fetal bovine serum (FBS; Gibco, USA) and maintained at 37 ^°^C in a humidified incubator with 5% CO_2_. When the cells reached approximately 80% confluence, they were digested with 0.25% trypsin for about 1 min. After removing the trypsin solution, the cells were counted and resuspended at a density of 1 × 10^5^ cells/mL, then seeded into appropriate culture plates for subsequent experiments. All experiments were performed with at least three independent biological replicates. Following trypsinization, the cells were counted and resuspended at a density of 1 × 10^5^ cells/mL. They were then seeded into appropriate culture plates and maintained in antibiotic-free RPMI 1640 medium. Once the cells reached 80–90% confluence, they were used for subsequent experiments. All assays were performed with at least three independent biological replicates.

The *STEC* strain CD15 H34 is a bovine derived Ounk:H4 serotype isolate carrying both *stx1a* and *stx2a* Shiga toxin genes and belonging to sequence type ST10 (Zhang et al., 2024). The strain was cultured in LB broth (Hopebio Co., Ltd., China) at 37 ^°^C with shaking at 180 rpm for 6 h.

### 2.3 *STEC* CD15-H34 infected HIEC

The *STEC* CD15-H34 strain was serially diluted tenfold to prepare bacterial suspensions ranging from 10^2^ to 10^9^ CFU/mL. Each dilution was inoculated onto HIEC monolayers that had reached 80–90% confluence. Infections were performed for 0.5, 1, 2, and 4 h, while control groups were treated with an equal volume of sterile medium under identical conditions. All experiments were carried out in triplicate using three independent biological replicates (*n* = 3). Following infection, cell viability was quantified using a Cell Counting Kit-8 (CCK-8) assay. In parallel, cell density and nuclear morphology were evaluated via DAPI staining. For each group, three randomly selected independent fields of view were captured under a fluorescence microscope, and DAPI-stained nuclei were counted using ImageJ software (NIH, USA). Cell density was expressed as the average number of nuclei per field, and the percentage reduction was calculated relative to the uninfected control. Transmission electron microscopy (TEM; Tecnai G2 F30 S-Twin, Philips-FEI, Netherlands) and scanning electron microscopy (SEM; SU8010, Hitachi, Japan) were employed to visualize the surface morphology of HIEC cells and bacterial adhesion patterns after infection.

### 2.4 miRNA and mRNA library construction

**Figure 1:**
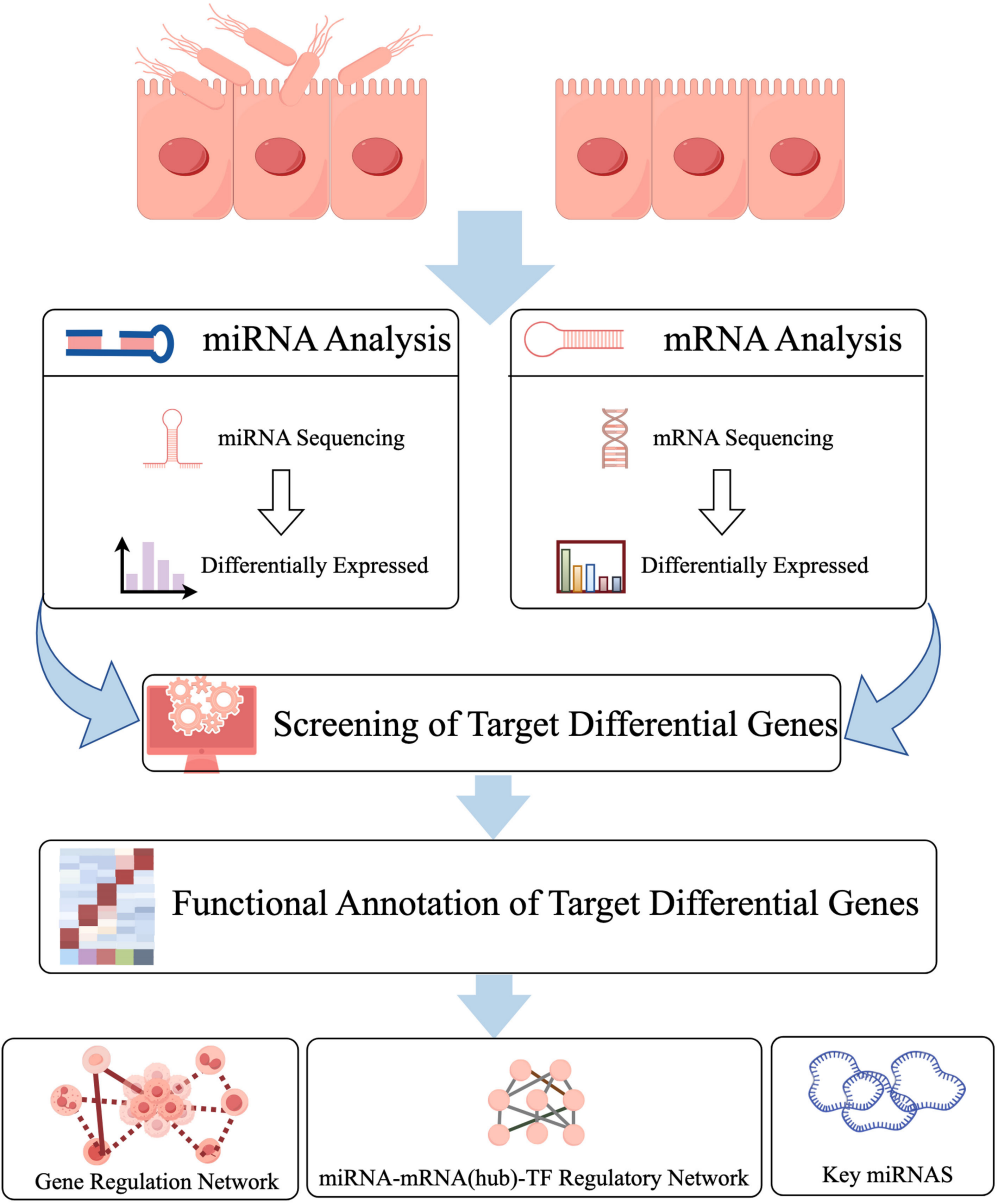
Flow chart of the experimental workflow.

Total RNA was extracted from *STEC*-infected (*n* = 3) and control (*n* = 3) HIEC cells using TRIzol reagent (Invitrogen, USA) via standard phenol-chloroform extraction, according to the manufacturer instructions (Wu et al., 2024). Extracted RNA was treated with DNase I (Ambion TURBO DNase, USA) to remove genomic DNA and purified using centrifugal columns. RNA purity was assessed with a NanoDrop 2000 spectrophotometer (Thermo Fisher Scientific, USA), concentration was measured with the Qubit RNA HS Assay Kit (Invitrogen, USA). RNA integrity was determined using the Agilent 2100 Bioanalyzer system with the RNA 6000 Nano Kit (Agilent Technologies, USA). Only RNA samples meeting the required quality criteria were used for downstream analyses. The bioinformatics workflow is illustrated in **Figure 1**.

Small RNA libraries were prepared using the NEBNext^®^ Multiplex Small RNA Library Prep Set for Illumina (New England Biolabs, USA), following the instructions provided by the manufacturer. The resulting libraries were enriched by PCR amplification, and the amplified products were purified using 15% polyacrylamide gel electrophoresis (PAGE). Purified libraries were evaluated for quality using the Agilent High Sensitivity DNA Kit and the Agilent 2100 Bioanalyzer (Agilent Technologies Inc., USA). Library concentrations were measured with the Quant iT™ PicoGreen^®^ dsDNA Assay Kit (Invitrogen, USA). Sequencing was performed on the Illumina HiSeq platform in single end mode. Following sequencing, quality-controlled clean reads were mapped to the human reference genome and the miRBase database to identify human miRNAs. To preclude potential bacterial contamination, the reads were also aligned against the *Escherichia coli* reference genome. Any reads mapping to the *Escherichia coli* genome, as well as those failing to align with the human references, were excluded from downstream analyses.

Total RNA was processed using the Epicentre Ribo-Zero™ rRNA Removal Kit (Human) (Illumina, USA) to deplete ribosomal RNA (rRNA). The purified RNA was randomly fragmented with divalent cations under ion-mediated cleavage conditions to generate fragments of approximately 300 bp. These RNA fragments were then used as templates for mRNA library construction. Library size distribution and total concentration were assessed using the Quantifluor-ST fluorometer (Promega, Madison, USA) and the Quant-iT™ PicoGreen^®^ dsDNA Assay Kit (Invitrogen, USA). Library quality was evaluated on the Agilent 2100 Bioanalyzer with the Agilent High Sensitivity DNA Kit (Agilent Technologies Inc., USA). The qualified libraries were subjected to high-throughput sequencing on the Illumina platform.

### 2.5 Database verification

Four differentially expressed miRNAs (miR-543, miR-370-3p, miR-33-3p, and miR-3065-5p) and four differentially expressed mRNAs (PIK3A, IL6, BBC3, and FOSL1) were randomly selected for qRT-PCR validation of the RNA-seq results (Zhang et al., 2024). Total RNA was extracted from infected and control HIEC cells using TRIzol Reagent (Invitrogen, USA). miRNAs and mRNAs were reverse-transcribed into cDNA using a commercial reverse transcriptase kit (TransGen Biotech, China) according to the manufacturer’s instructions. qPCR was carried out with a SYBR Green qPCR kit (TransGen Biotech, China) on an Applied Biosystems 7500 Fast Real-Time PCR System (Applied Biosystems, USA), using GAPDH as the reference gene. Relative expression levels were calculated by the 2^−ΔΔ^*^CT^* method (Fan et al., 2021). Primer sequences for both mRNAs and miRNAs are listed in **Supplementary Table 1**.

### 2.6 Screening of differentially expressed miRNAs and differentially expressed mRNAs

The gene expression levels were normalized using the FPKM method (Fragments Per Kilo bases per Million fragments). Differentially expressed miRNAs and mRNAs were analyzed using DESeq2 (version 1.38.3). Differentially expressed and conserved miRNAs and mRNAs were identified based on the criteria of |log_2_ fold change| *>* 1 and *P <* 0.05 (Anders and Huber, 2010). Volcano plots of differentially expressed miRNAs were generated using the R programming language (v4.3.0) and the ggplot2 package.

### 2.7 Differentially expressed miRNA target gene screening and functional annotation

The target mRNAs of up-regulated and down-regulated differentially expressed miRNAs were predicted using miRanda (http://mirtoolsgallery.tech/). Candidate target mRNAs were defined as those genes present in both the predicted target set and the list of differentially expressed mRNAs. Venn diagrams were generated using HiPlot (https://hiplot.com.cn/cloud-tool/drawing-tool/detail/113), and these overlapping targets were subjected to subsequent analyses.

### 2.8 GO and KEGG analyses

GO annotation and KEGG pathway enrichment analyses of the upregulated and downregulated target mRNAs were performed using the Database for Annotation, Visualization, and Integrated Discovery (DAVID, https://davidbioinformatics.nih.gov/) Terms and pathways with *P <* 0.05 were considered statistically significant (Huang et al., 2009; Géczi et al., 2025).

### 2.9 PPI network construction

To analyze interactions among the proteins encoded by differentially expressed genes (DEGs), a protein-protein interaction (PPI) network was constructed using the STRING database (http://string-db.org/). The minimum required interaction score was set to 0.4 (medium confidence), with all other parameters maintained at default settings (Szklarczyk et al., 2019). The network was then analyzed using the cytoHubba plugin in Cytoscape. Node centrality was calculated using the Maximum Clique Centrality (MCC) algorithm. The top 10% of genes by MCC score were selected for downstream analysis, with the ten highest-scoring genes explicitly defined as hub genes; their corresponding transcripts were thus designated as hub mRNAs. Subsequently, a hierarchical regulatory network was established positioning these hub mRNAs as core targets. Within this framework, upstream miRNAs and TFs, we mapped the complete landscape of both post-transcriptional and transcriptional regulation (Gao et al., 2022).

### 2.10 Construction of miRNA–hub mRNA–TF gene regulatory networks and identification of key miRNAs

Upstream transcription factors (TFs) of hub genes were predicted using TRRUST v2.0 and hTFtarget (https://guolab.wchscu.cn/hTFtarget/) (Zhao et al., 2023; Zhang et al., 2020). TFs identified by both databases were considered high confidence candidates. A TF–hub mRNA regulatory network was visualized in Cytoscape (v3.9.1) and integrated with predicted miRNA–hub mRNA interactions to construct a comprehensive miRNA–hub mRNA–TF regulatory network, revealing potential multi-level post transcriptional regulation (Wang et al., 2022). Using the CytoNCA plugin in Cytoscape (v3.9.1), the top three miRNAs with the highest number of target genes were identified based on network topology parameters, including Betweenness Centrality (BC), Eigenvector Centrality (EC), and Information Content (IC). These miRNAs were subsequently defined as key regulatory miRNAs (Huang et al., 2021; Zhang et al., 2022; Chen et al., 2020).

### 2.11 Statistical analysis

Statistical analyses were performed using SPSS version 27. Data are presented as mean ± standard deviation (SD). Differences between two groups were assessed by independent samples *t*-test. Two tailed *P*-values of *<* 0.05, were considered to indicate statistically significant (Liu et al., 2023).

## 3 Results

### 3.1 Effects of *STEC* CD15-H34 infection on viability and morphology of HIEC

**Figure 2:**
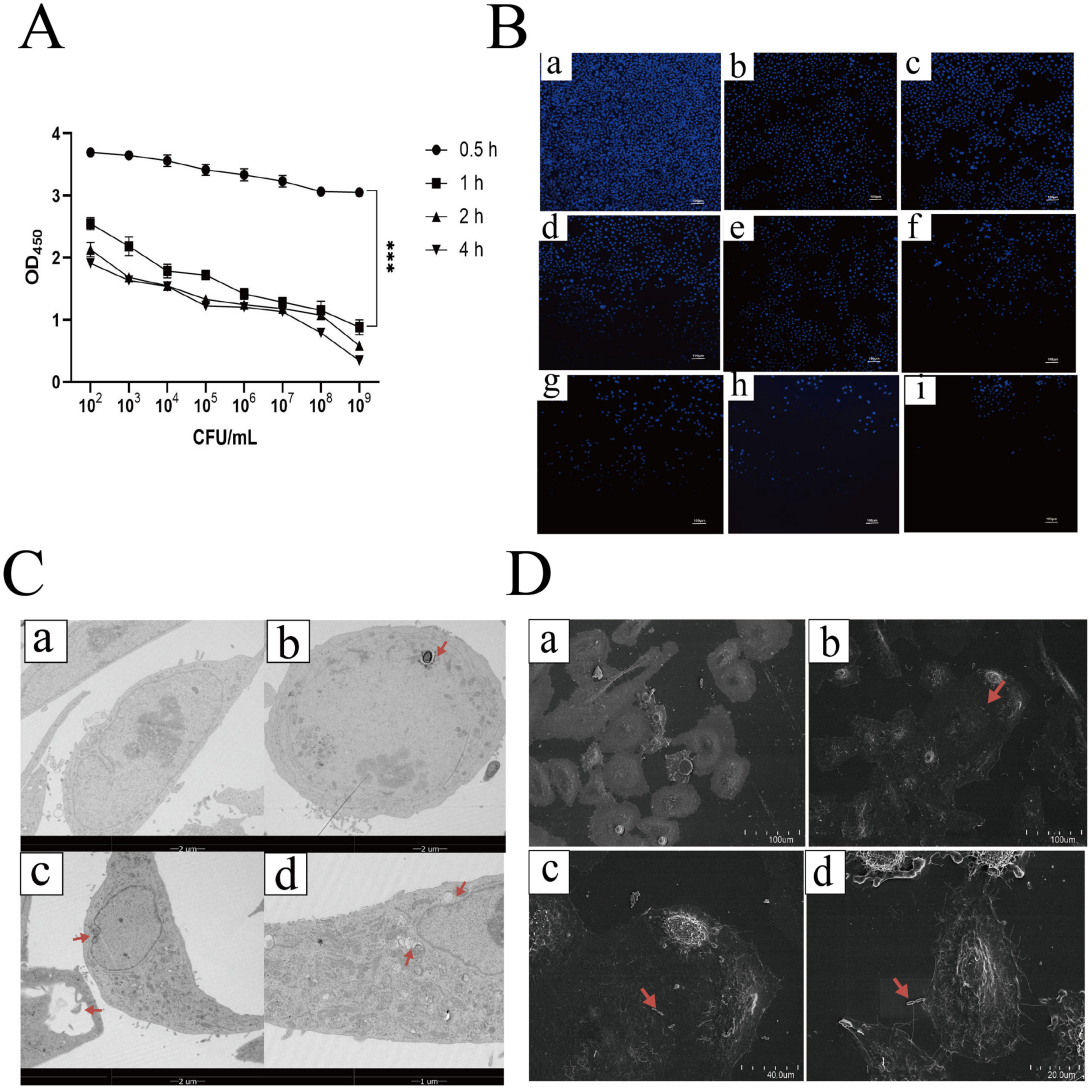
The bacteria count and timing of *STEC* CD15-H34 infection in HIECs. **(A)** CCK8 assayed the cellular viability of HIEC infected with *STEC* for 0.5 h, 1 h, 2 h and 4 h at different concentrations (10^2^ CFU/mL to 10^9^ CFU/mL), respectively. The data are presented as mean ± SD with a sample size of *n* = 3. Statistical analyses revealed significant differences, with ** indicating *p <* 0.01 and *** indicating *p <* 0.001. **(B)** HIEC were treated with *STEC* at concentrations ranging from 10^2^ to 10^9^ CFU/mL for 1 h, followed by DAPI staining. In this context, a represents the control group, while b, c, d, e, f, g, h, and i correspond to infected groups with concentrations of 10^2^, 10^3^, 10^4^, 10^5^, 10^6^, 10^7^, 10^8^, and 10^9^ CFU/mL, respectively. **(C)** HIEC were infected with 10^6^ CFU/mL of *STEC* for 1 h and subsequently observed by TEM. (a) at 1700× magnification, displays uninfected HIEC with a clearly defined cell membrane encasing the entire cell. The nuclear structure is intact and normal, the cytoplasm is evenly distributed, and the mitochondria exhibit normal morphology. (b) also at 1700× magnification, reveals the absence of typical nuclear structure within the cells, with swollen mitochondria and loss of cristae. (c) at 1700× magnification, shows the formation of large vacuoles within the cells, with no distinct structure inside the vacuoles, an increase in granular material in the cytoplasm resulting in turbidity, and the disappearance of mitochondrial cristae. (d) at 3500× magnification, demonstrates indentation of the cell membrane, expansion and deformation of the endoplasmic reticulum, mitochondrial swelling or disintegration of cristae, and the presence of autophagosomes or autophagic vacuoles. **(D)** HIEC were infected with 10^6^ CFU/mL of *STEC* for 1 h and subsequently observed by SEM. (a) at 500× magnification, depicts uninfected HIEC with tightly packed cells and normal intercellular connections. The surface structure is clearly visible, exhibiting natural protrusions and tight junctions. (b) also at 500× magnification, shows a decreased number of cells with loosened intercellular connections. Rod-shaped bacteria are observed adhering to the cell surface, which presents indentations and a rough, irregular morphology. (c) at 1300× magnification, reveals disrupted or even broken intercellular connections, bacteria adhered to the cell surface, and distinct protrusions or indentations in the cell outline, with a rough surface. (d) at 2200× magnification, demonstrates cell fragmentation, bacteria adhering to the cell surface, an irregular cell outline, the presence of small protrusions and fragments on the surface, and damage to the cell membrane.

To evaluate the infectivity of the STEC CD15-H34 strain in HIEC cells, we employed a CCK-8 assay to assess the impact of varying bacterial inocula (10^2–^10^9^ CFU/mL) and infection durations on cell viability. The results demonstrated that the inhibition of HIEC viability by STEC CD15-H34 was strongly dictated by both the bacterial concentration and the duration of exposure. Specifically, cell viability declined progressively as the bacterial load increased. Furthermore, prolonged infection exacerbated this cellular damage, with viability at 1 h being significantly lower than that at 0.5 h (*p <* 0.001) (**Figure 2A**). Specifically, infection with 10^6^ CFU/mL for 1 h resulted in a cell viability of approximately 44.4%. To validate these findings, DAPI staining and nuclear counting were performed. The staining results aligned with the CCK-8 data, revealing a gradient reduction in cell density as the infection dose increased. At 10^6^ CFU/mL for 1 h, cell density was reduced by approximately 50% relative to the control group. We selected this condition for subsequent analyses (**Figure 2B**).

Further examination of the ultrastructure and surface morphology of HIEC infected with 10^6^ CFU/mL *STEC* CD15-H34 was performed using TEM and SEM. TEM revealed discontinuities in the cell membrane 1 h after *STEC* infection, accompanied by pronounced disruption of organelle structures and the presence of numerous cytoplasmic vacuoles. Some cells exhibited nuclear fragmentation or complete nuclear loss (**Figure 2C**). SEM analysis showed that portions of the infected cell population displayed bacterial adherence, surface collapse, wrinkling, and rupture, as well as incomplete membrane margins and cytoplasmic condensation (**Figure 2D**). Infection of HIEC with 10^6^ CFU/mL *STEC* CD15-H34 for 1 h markedly reduced cellular viability, caused severe disruption of nuclear and organelle architecture, and induced pronounced alterations in cell surface morphology, indicating loss of structural integrity and impaired cellular function. Together, these findings show that *STEC* CD15-H34 exerts cytotoxic effects on HIEC, leading to pronounced cellular stress and cytotoxicity. Taken together, these results demonstrate that STEC CD15-H34 infection significantly reduces HIEC viability, disrupts nuclear and organelle integrity, and causes morphological aberrations at the cell surface, indicative of a profound state of cellular stress and dysfunction. To further elucidate the molecular mechanisms underlying these phenotypic alterations, we subsequently performed RNA-seq analysis.

### 3.2 Differential expression profiling of miRNAs and mRNAs in *STEC* infected HIEC

**Figure 3:**
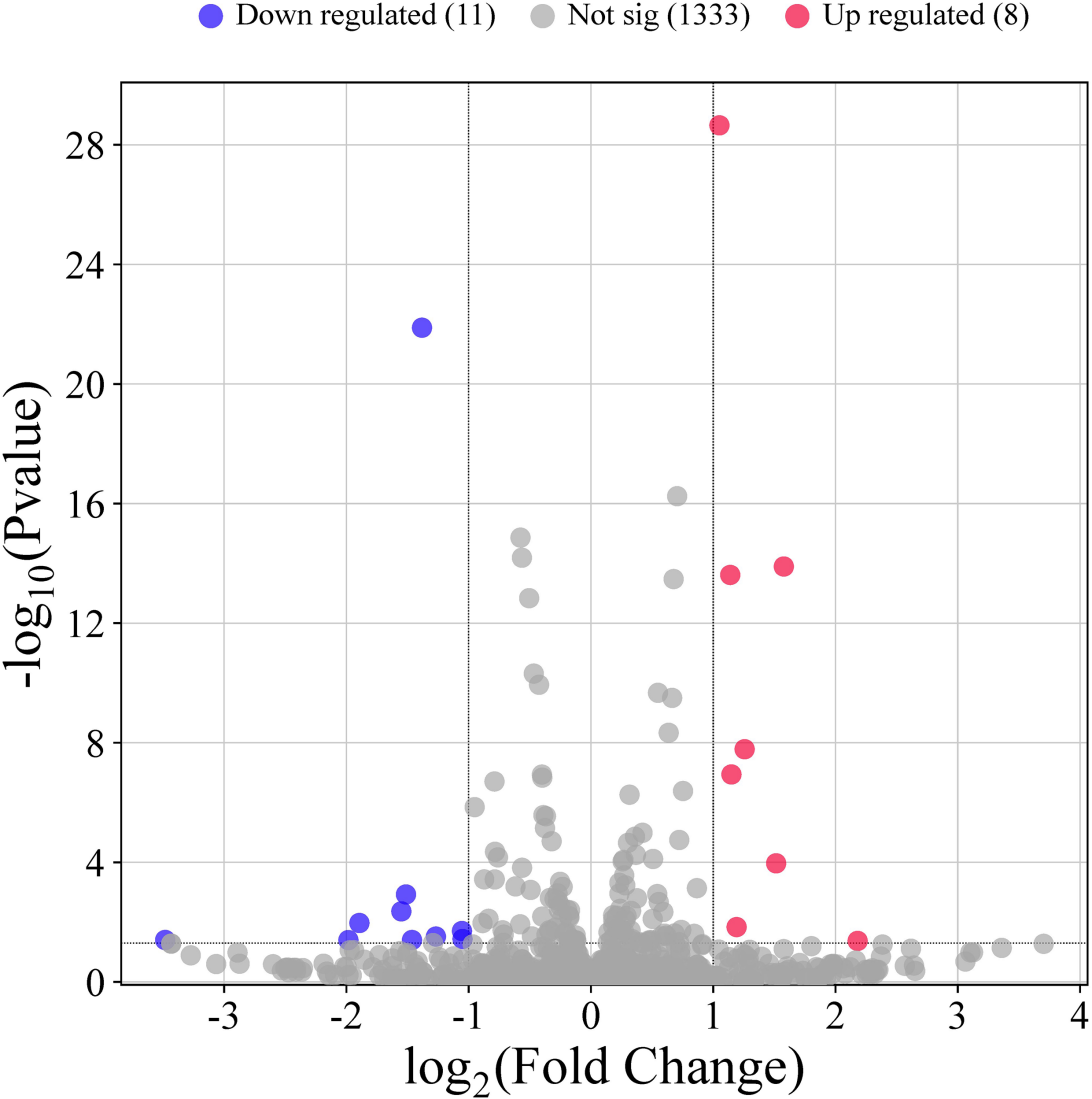
Screening of differentially expressed miRNAs of the RNA-seq database. Volcano plot illustrating the differential expression of miRNAs between the infected group (H1, H2, H3) and the control group (K1, K2, K3).

To identify miRNAs and mRNAs induced by *STEC* CD15-H34 infection, small RNA-seq and mRNA-seq data were generated from control HIEC and HIEC infected with 10^6^ CFU/mL of the *STEC* CD15-H34 for 1 h. To systematically elucidate the post-transcriptional regulatory mechanisms in HIEC cells during STEC infection, we analyzed miRNA and mRNA sequencing data from uninfected controls and cells infected with the STEC CD15-H34 strain (10^6^ CFU/mL for 1 h). Illumina sequencing yielded an average of 8.75 million small RNA reads per sample. Following the filtration of low-quality tags, 3^′^ adaptors, contaminants, and non-target non-coding RNAs, approximately 3.2 million unique high-quality reads (18–36 nt) were retained across all samples for downstream analysis. Genome alignment identified 547 known miRNAs and 1,724 novel miRNAs (**Table 1**). Differential expression analysis revealed 19 miRNAs with significant changes between the infected and control groups, including 8 upregulated and 11 downregulated miRNAs (**Figure 3**).

**Table 1:** Sequencing and analysis of small RNA libraries from the *STEC*-infected and control groups.

Sample	Total reads	Clean reads	Unique reads	Identified known miRNAs	Identified novel miRNAs
H1	14,681,376	8,571,594	629,405	886	333
H2	16,278,045	9,141,235	664,136	919	361
H3	13,814,226	8,442,472	610,916	907	326
K1	14,103,016	8,154,366	450,326	925	203
K2	13,953,633	7,511,458	438,663	897	228
K3	14,717,421	8,765,098	483,991	943	273

Total reads: The number of sequences in the raw data. Clean reads: The number of sequences with a nucleotide length ≥ 18 nt. Unique reads: The number of unique Small RNA sequences in a single sample. Identified known miRNAs: The number of mature miRNAs successfully identified in the sequencing data after comparison with a known miRNA database. Identified novel miRNAs: The number of mature miRNAs identified in the sequencing data that have not yet been recorded in the known miRNA database.

**Figure 4:**
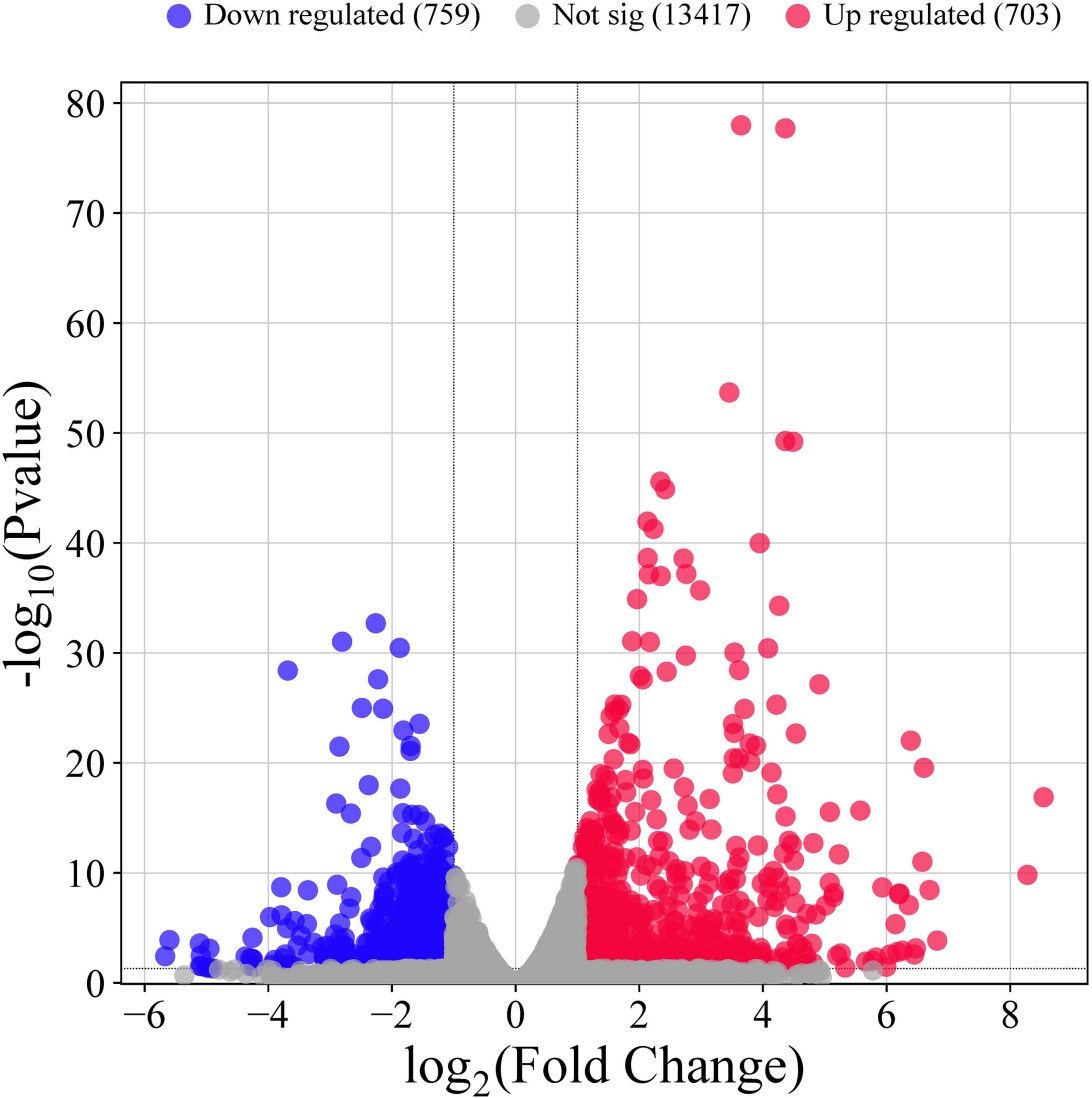
Screening of differentially expressed mRNAs of the RNA-seq database. Volcano plot depicting the differential expression of mRNAs in the infected group (H1, H2, H3) and the control group (K1, K2, K3).

For mRNA sequencing, approximately 77.19 million reads were obtained per group, with more than 95% classified as clean reads (740,895,188 in total). Quality assessment showed that both groups exhibited Q20 and Q30 values exceeding 98.22% and 95.12%, respectively, confirming the high quality of the data suitable for downstream analyses **Table 2**. In total, 1,462 differentially expressed mRNAs were identified between the infected and control groups, comprising 703 upregulated and 759 downregulated transcripts (**Figure 4**).

**Table 2:** mRNA library sequencing and analysis in the *STEC* infected group and the control group.

Sample	Total reads	Clean reads	Clean reads (%)	Q20 (%)	Q30 (%)
H1	135,389,576	111,272,636	82.18	98.29	95.12
H2	135,749,008	111,122,814	81.85	98.40	95.36
H3	114,459,832	92,421,962	80.74	98.39	95.38
K1	132,901,322	112,626,122	84.74	98.27	95.20
K2	129,820,128	104,241,208	80.29	98.22	95.19
K3	123,632,786	100,750,678	81.49	98.48	95.64

Total reads: The total number of sequences associated with mRNA. Clean reads: The number of high-quality sequence reads. Clean reads (%): The proportion of high-quality sequence reads compared to the total number of sequencing reads. Q20 (%): The percentage of bases exhibiting a base call accuracy exceeding 99%. Q30 (%): The percentage of bases exhibiting a base call accuracy exceeding 99.9%.

### 3.3 qRT-PCR validation confirms RNA-Seq findings

**Figure 5:**
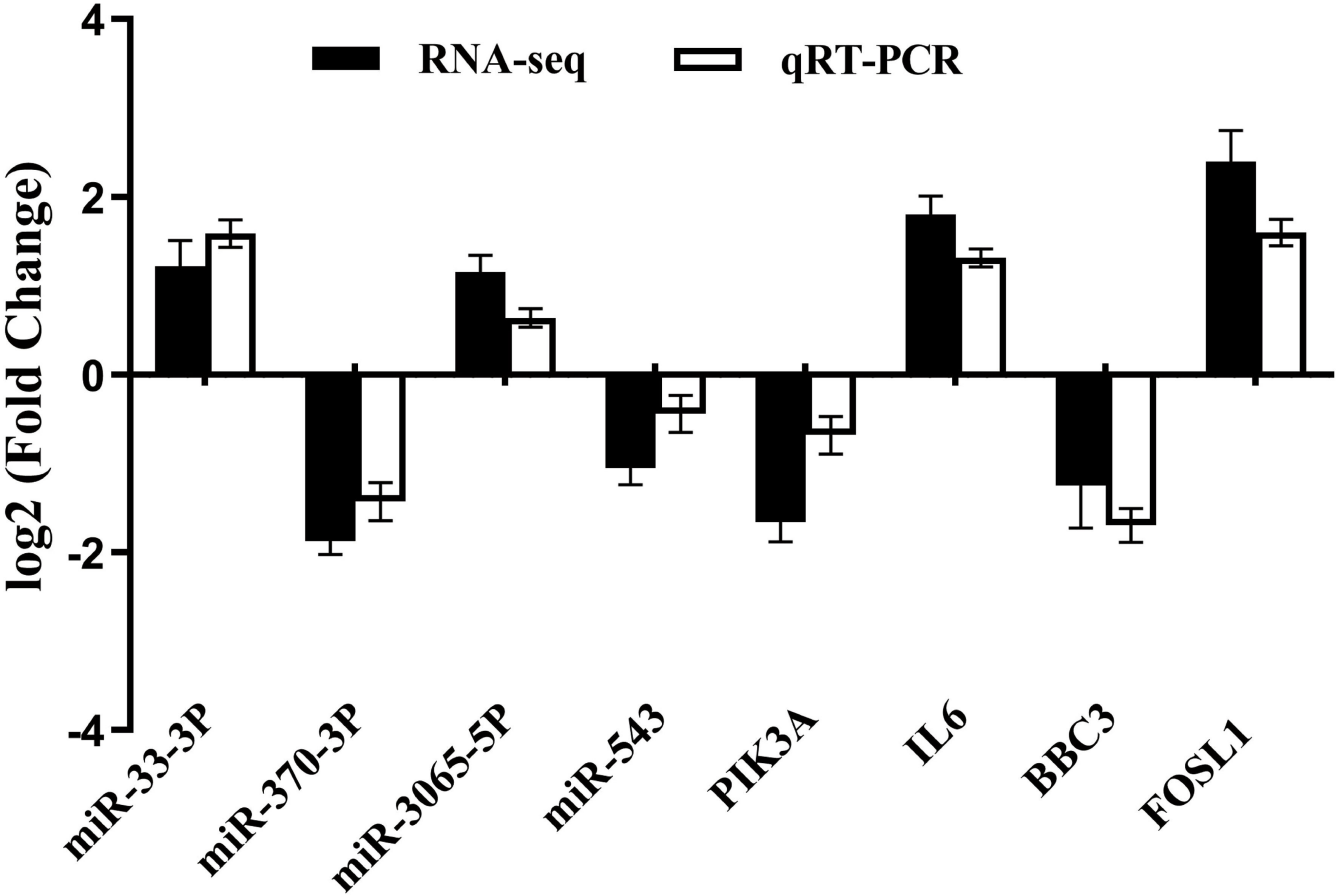
Comparison of the expression levels of 4 differentially expressed miRNAs and 4 differentially expressed mRNAs as determined by qRT-PCR and RNA-seq methods. The fold change was determined by normalizing to GAPDH using the 2^−ΔΔ^*^Ct^* method. The qRT-PCR data were shown as mean ± SD with a sample size of *n* = 3.

To validate the RNA-seq results, four differentially expressed miRNAs (miR-543, miR-370-3p, miR-33b-3p, miR-3065-5p) and four differentially expressed mRNAs (PIK3A, IL6, BBC3, and FOSL1) were selected for quantitative real-time PCR (qRT-PCR). qRT-PCR confirmed that the expression patterns of these transcripts were highly consistent with the RNA-seq data (**Figure 5**), supporting the accuracy and reproducibility of the transcriptomic dataset.

### 3.4 Screening and functional annotation of target genes for differentially expressed miRNAs

**Figure 6:**
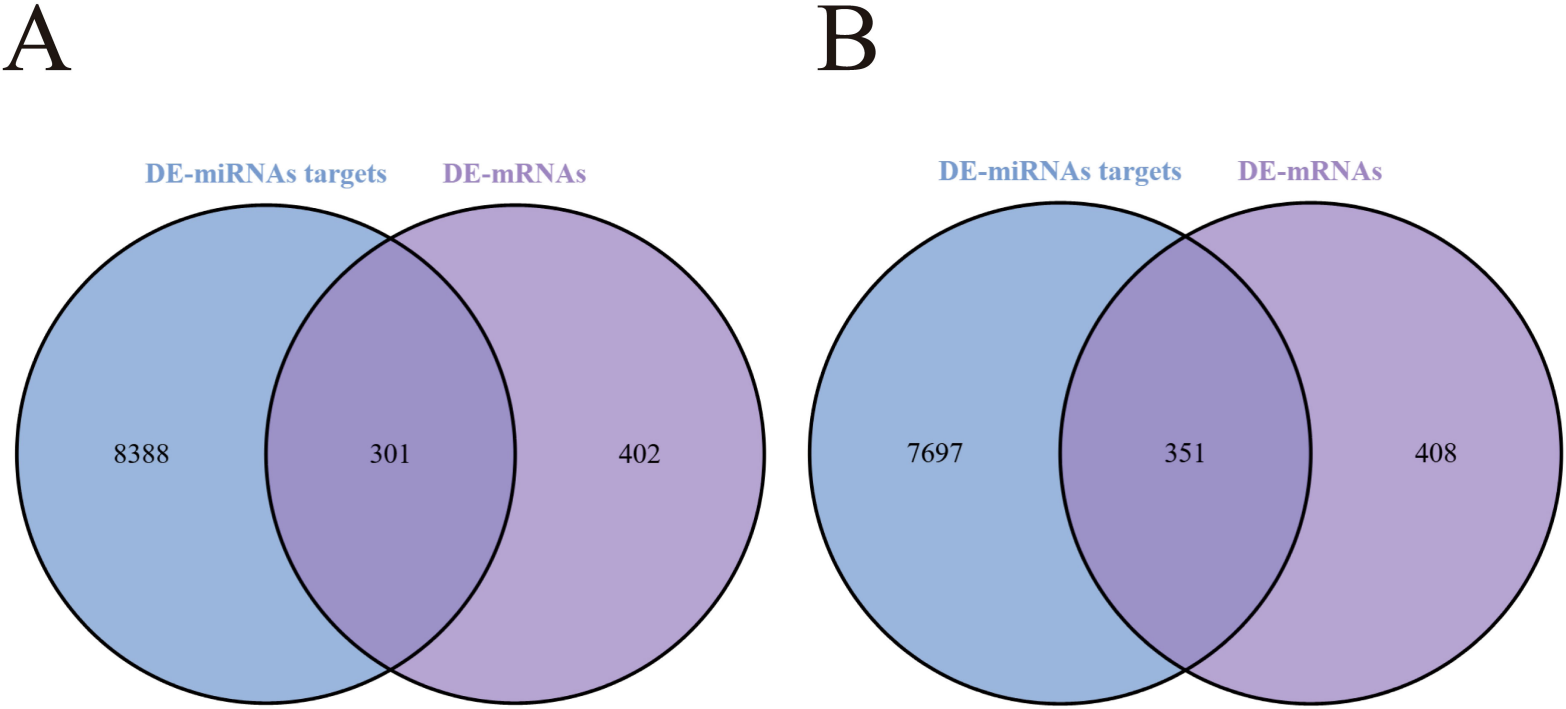
Screening of differentially expressed target mRNAs regulated by miRNAs in *STEC* infected HIEC. **(A)** Screening of upregulated target mRNAs. **(B)** Screening of downregulated target mRNAs.

To elucidate miRNA regulatory roles during *STEC* CD15-H34 infection, prediction of targets in cis was performed for the 19 differentially expressed miRNAs, yielding 11,271 putative target mRNAs. The 8 upregulated miRNAs were predicted to target 8,048 mRNAs, and the 11 downregulated miRNAs were predicted to target 8,689 mRNAs. Overlap analysis between predicted targets and the differentially expressed mRNAs from RNA-seq identified 351 predicted targets that were downregulated **Figure 6A**), and 301 predicted targets that were upregulated in infected cells (**Figure 6B**). These findings suggest that, during *STEC* infection, differentially expressed miRNAs engage in close regulatory interactions with their target mRNAs, indicating that miRNAs are likely involved in the host cellular response to infection by modulating specific mRNA networks.

### 3.5 Analysis of differentially expressed miRNAs and mRNAs

**Figure 7:**
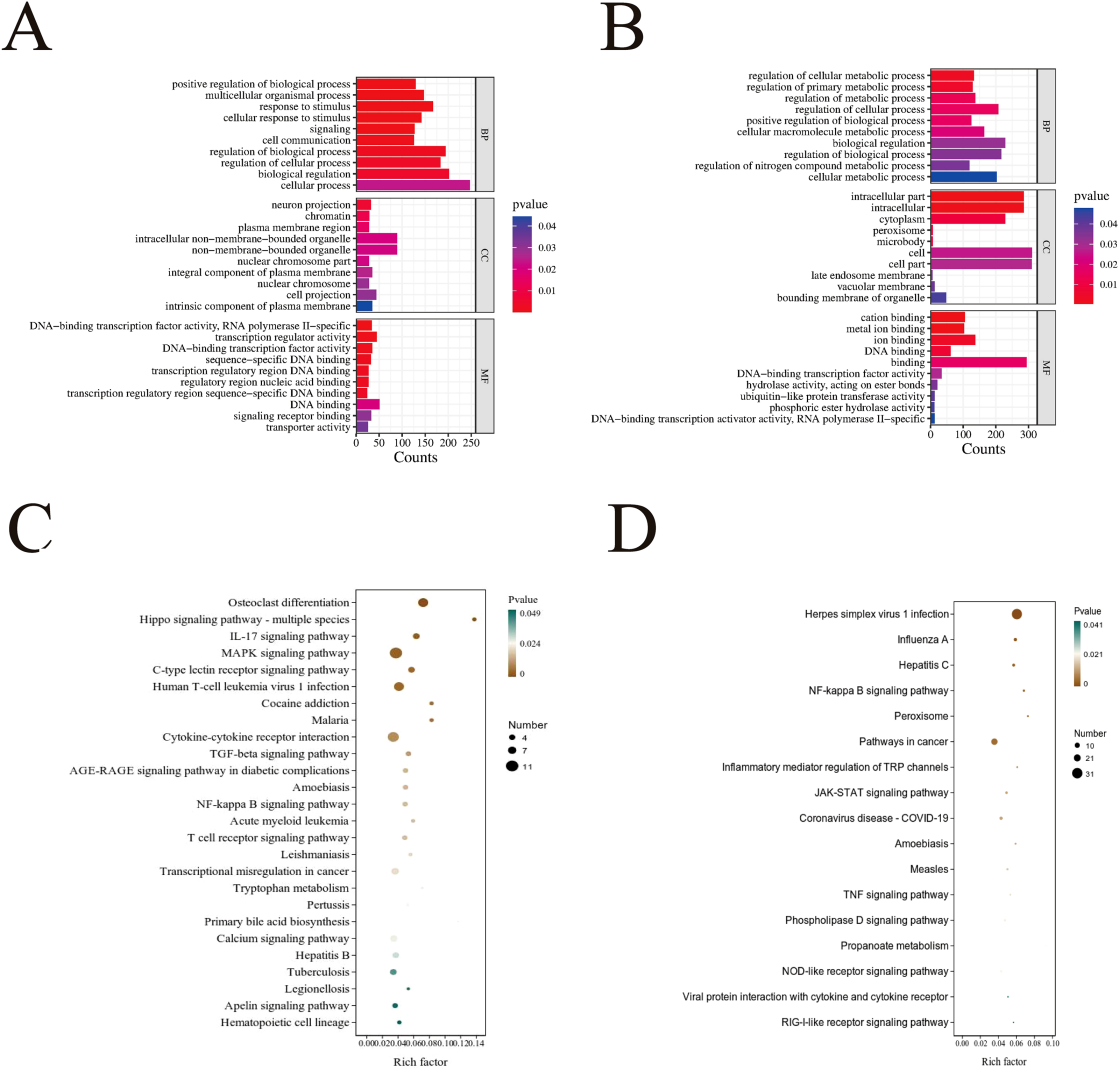
GO and KEGG enrichment analyses of upregulated and downregulated target mRNAs. **(A)** GO enrichment analysis of upregulated target mRNAs. **(B)** GO enrichment analysis of downregulated target mRNAs. **(C)** KEGG enrichment analysis of upregulated target mRNAs. **(D)** KEGG enrichment analysis of downregulated target mRNAs. The size of the bubble indicates the number of enriched target genes in that pathway, while the color of the bubble represents different ranges of *P* values. The Rich factor is the ratio of the number of annotated differential genes in that pathway to the total number of annotated genes in that pathway.

To understand the biological functions of the differentially expressed target genes, we conducted GO and KEGG pathway enrichment analyses for both upregulated and downregulated target mRNAs. GO enrichment analysis revealed that target mRNAs were distributed across a broad spectrum of biological categories, indicating that STEC infection triggers a systemic and multi-layered regulation of host cell functions. Specifically, upregulated targets were significantly enriched in pathways associated with host defense and signal transduction, such as response to stimulus and cell communication. The prominent enrichment of transporter activity and integral component of plasma membrane further suggests a reconfiguration of the cell surface interface. In contrast, downregulated targets were primarily concentrated in metabolic regulation pathways, including cellular metabolic process and nitrogen compound metabolic process, as well as enzymatic activities like ion binding. This distinct functional separation implies that STEC infection induces metabolic reprogramming, where host cells appear to suppress basal metabolic activities to reallocate resources toward signal transduction and immune defense mechanisms. Notably, while the top three enriched pathways for upregulated and downregulated genes overlapped, their specific sub-categories exhibited clear functional divergence. This suggests that target mRNAs may participate in core biological processes through compensatory regulation, simultaneously orchestrating HIEC biological behavior at multiple levels. The top 10 enriched functional subclasses for upregulated and downregulated target mRNAs in BP, CC, and MF are visualized respectively (**Figures 7A, B**).

To further understand the specific regulatory functions of the target mRNA, we conducted KEGG analysis on the upregulated and downregulated target mRNAs to explore their enrichment in five pathways: cellular processes, environmental information processing, genetic information processing, human diseases, and organismal systems. The results showed that the upregulated target mRNAs were involved in 211 pathways, while the downregulated target mRNAs were involved in 242 pathways. A bubble chart was used to display the 26 pathways significantly enriched in upregulated target mRNAs (**Figure 7C**) and the 17 pathways significantly enriched in downregulated target mRNAs (**Figure 7D**). Specifically, the upregulated target genes were primarily enriched in the MAPK signaling pathway and the cytokine-cytokine receptor interaction pathway. In contrast, the downregulated differentially expressed genes were mainly enriched in the herpes simplex virus 1 infection and pathways in cancer. This indicates that the differentially expressed target mRNAs play a multidimensional role in regulating cellular stress responses and immune regulation, contributing to the maintenance of basic cellular structure and related disease pathways.

### 3.6 Construction of the PPI regulatory network

**Figure 8:**
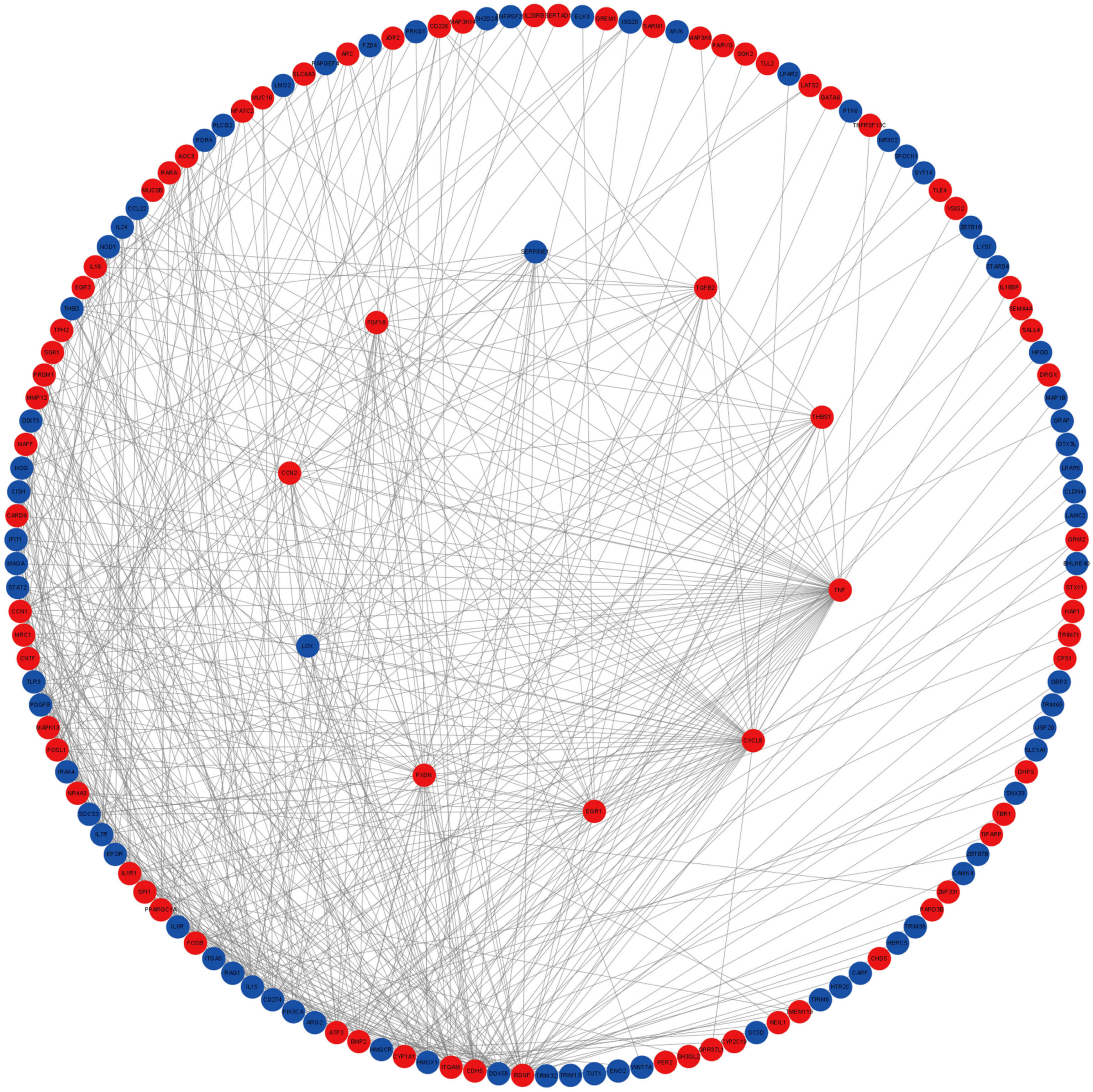
PPI network of differentially expressed target genes in *STEC* Infected HIEC. PPI network of differentially expressed genes. The inner circle represents the key mRNAs identified through screening, with red nodes indicating upregulated genes and blue nodes indicating downregulated genes.

To further explore the potential functions of differentially expressed target mRNAs during *STEC* infection of HIEC and their roles in molecular regulation, we employed the STRING database to analyze PPI relationships among the proteins encoded by these mRNAs. This analysis yielded a PPI network comprising 639 nodes and 1,098 interaction edges (**Supplementary Figure 1**). Subsequent visualization of the top 10% (65) key proteins in the network using CytoHubba (**Figure 8**) identified 10 hub genes TNF, CXCL8, CCN2, SERPINE1, THBS1, CDH5, PXDN, TGFB2, LOX, and FGF18 which may serve as central regulators during *STEC* infection of HIEC.

### 3.7 Construction of the miRNA–mRNA–TF gene regulatory network

To elucidate the functions and potential mechanisms of the identified miRNAs, we constructed a miRNA–TF–mRNA regulatory network. TRRUST v2 predictions identified 14 TFs corresponding to 10 hub genes (**Supplementary Table 2**), while hTFtarget predicted 208 TFs associated with the same 10 hub genes (**Supplementary Table 3**). The intersection of the two datasets comprised 11 TFs, NR4A1, E2F1, ERG, ATF2, USF2, RELA, NFKB1, CEBPB, USF1, JUN and SP1.

**Figure 9:**
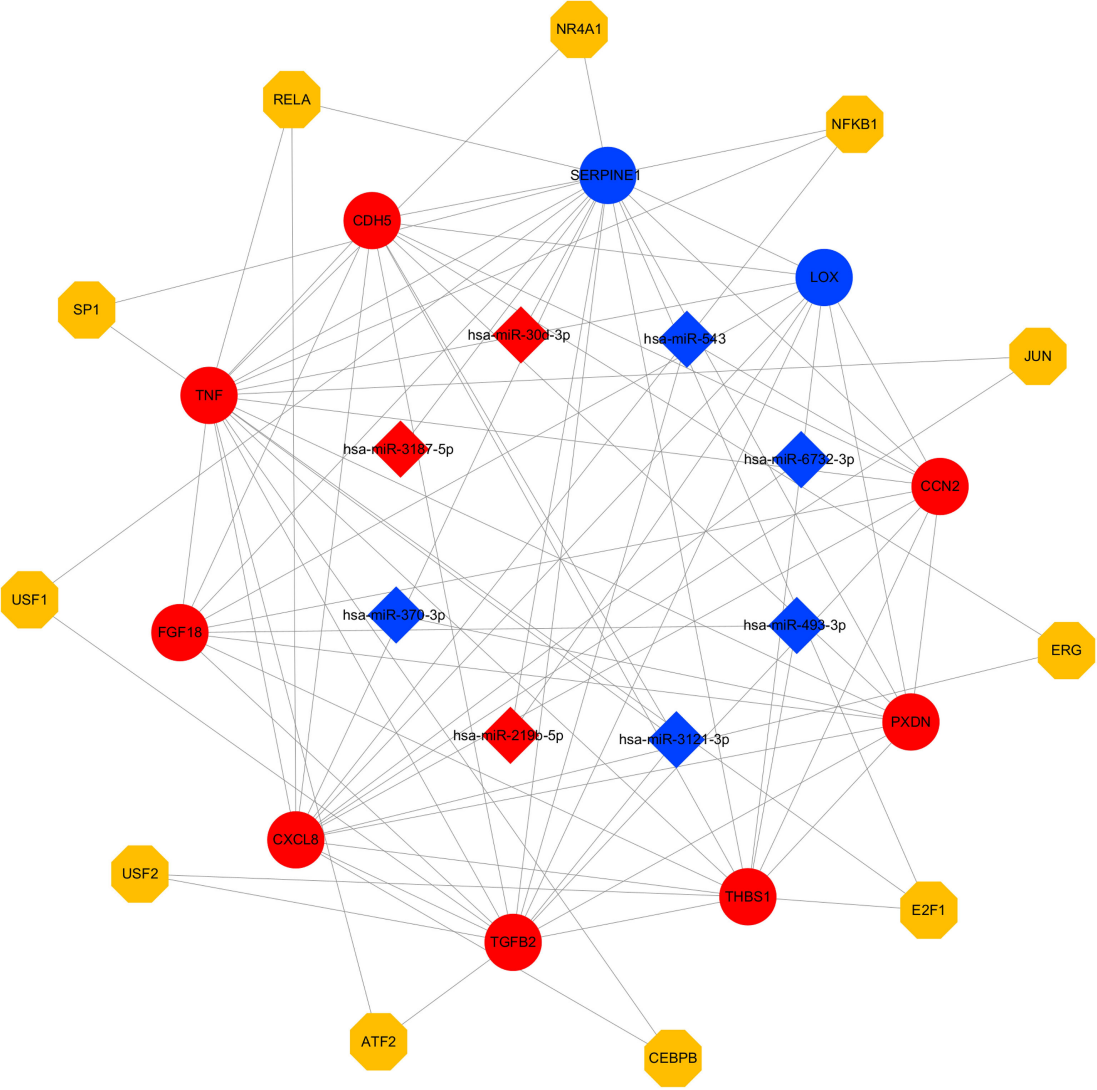
miRNA–Hub mRNA–TF regulatory network of differentially expressed target genes in *STEC-*Infected HIEC. Squares denote miRNAs, circles denote mRNAs, and diamonds denote TF genes. Red and blue nodes indicate upregulated and downregulated genes, respectively, while yellow nodes represent TFs.

Based on the miRNA–mRNA network, a miRNA–mRNA–TF regulatory network of 29 nodes (8 miRNAs, 10 mRNAs, 11 TFs) and 82 edges was constructed in Cytoscape (**Figure 9**). Network topology analysis identified miR-3121-3p, miR-219b-5p, and miR-543 as hub miRNAs.

## 4 Discussion

In this study, we systematically identified differentially expressed miRNAs in *STEC* infected HIEC cells, constructed a miRNA–mRNA–TF regulatory network, and identified miR-3121-3p, miR-219b-5p, and miR-543 as potential key regulators of *STEC* infection in HIEC cells. Taken together, these findings identify specific molecular targets and provide a mechanistic foundation for early diagnosis, precision therapy, and targeted intervention of *STEC* infections.

One hour after *STEC* infection, the bacteria adhered to the surface of HIEC cells and caused noticeable cellular damage. This early adhesion represents a crucial prerequisite for the onset of *STEC* induced human disease (Detzner et al., 2022). Previous studies have demonstrated that *STEC* infection alters multiple physiological and molecular processes, including intestinal protein turnover, microbiota composition epithelial barrier integrity, and immune responses (Song et al., 2022). In this study, 19 differentially expressed miRNAs and their 652 target mRNAs were identified in *STEC* infected HIEC cells. GO enrichment analysis revealed that these target genes are primarily associated with the disruption of cellular structural integrity. This transcriptomic profile closely corroborates the morphological aberrations observed via SEM, specifically the membrane roughness, surface depressions, and compromised intercellular junctions. Furthermore, KEGG pathway analysis indicated that the upregulated target mRNAs were significantly enriched in core inflammatory and stress cascades, particularly the MAPK signaling pathway and cytokine-cytokine receptor interactions. (Plotnikov et al., 2011; Zhou et al., 2019). The miRNA-mediated hyperactivation of these pathways likely precipitates profound intracellular stress, culminating in the severe organelle damage observed under TEM. Collectively, these results suggest that the differentially expressed miRNAs may contribute to STEC induced cellular structural injury.

We constructed a miRNA–hub mRNA–TF network to uncover the transcriptional mechanisms driving STEC infection in HIEC cells. The reliability of these predictions was enhanced by intersecting target data from the TRRUST 2.0 and hTFtarget databases.citephan2018trrust (Han et al., 2018; Zhang et al., 2020). We identified 11 TFs exhibiting high-confidence regulatory interactions with 10 hub genes. Notably, NFKB1, RELA, and ATF2 emerged as prominent mediators of inflammation and cellular stress responses (Zhang et al., 2020; Song et al., 2025) (Watson et al., 2017). Specifically, NFKB1 and RELA, the core subunits of the NF-*κ*B signaling cascade, are known to amplify tissue pathology by driving the expression of pro-inflammatory cytokines such as TNF (Baker et al., 2011; Liang et al., 2004). Strikingly, our network predicted that hsa-miR-3121-3p and NFKB1 co-regulate TNF. This suggests a synergistic paradigm where TFs and miRNAs cooperatively orchestrate the expression of pro-inflammatory genes at both transcriptional and post-transcriptional levels, thereby dictating the trajectory of immune and inflammatory responses during STEC infection. Further *in vitro* and *in vivo* studies are required to validate this regulatory axis.

We identified miR-3121-3p, miR-219b-5p, and miR-543 as key regulatory factors during *STEC* infection of HIEC cells. These miRNAs may play critical roles in modulating infection-induced signaling pathways and immune responses, holding great promise as valuable therapeutic targets against *STEC* infection. Previous studies have shown that miR-3121-3p is involved in immune regulation, angiogenesis, and hormonal balance. It promotes cell migration, invasion, and proliferation by inhibiting Rap1GAP, thereby activating the MAPK signaling pathway (Qin et al., 2025; Xu et al., 2020). In this study, downregulation of miR-3121-3p was accompanied by the upregulation of inflammatory and adhesion/permeability related genes, including TGFB2, TNF, and CDH5. This finding suggests that reduced miR-3121-3p expression may enhance inflammatory responses and impair epithelial homeostasis by relieving suppression of its target genes. Furthermore, our analysis identified CCN2 and TGFB2 as putative targets of miR-543, a well-established regulator in diverse pathological conditions (Wang et al., 2023). As a pivotal member of the TGF-*β* superfamily, TGFB2 orchestrates critical cellular functions, including proliferation, migration, and immune modulation (Zheng et al., 2024). Previous studies have demonstrated that the transcription factor ATF2 upregulates TGFB2 via the MAPK signaling pathway (Zheng et al., 2024). Integrating these findings, we postulate that during STEC infection, miR-543 dysregulation intersects with these kinase cascades to dynamically fine-tune the host immune response. Conversely, we observed a marked upregulation of miR-219b-5p. Given its documented role in suppressing inflammatory mediators and mitigating tissue damage, this elevated expression likely reflects a compensatory host defense mechanism aimed at curbing excessive inflammation and facilitating tissue repair (Li et al., 2020);?. Taken together, the concurrent downregulation of miR-3121-3p and miR-543 likely synergizes to drive the aberrant activation of the TGF-*β* signaling pathway, while the upregulation of miR-219b-5p appears to serve as a compensatory host mechanism aimed at attenuating the ensuing inflammatory cascade. This dynamic crosstalk fundamentally dictates the pathological progression of *STEC* infection in HIEC cells, presenting a complex regulatory network that warrants future experimental validation.

Although the miRNA–mRNA–TF regulatory network constructed in this study provides valuable insights into the molecular mechanisms of *STEC* infection in HIEC cells and aids in identifying key regulatory targets, several limitations must be acknowledged. Our conclusions are primarily derived from transcriptomic sequencing data, which can reveal correlations but not establish causality. Therefore, the proposed regulatory mechanisms require rigorous experimental validation.

## Data Availability

The datasets presented in this study can be found in online repositories. The names of the repository/repositories and accession number(s) can be found in the article/**Supplementary Material**.

## References

[B1] Alharbi M. G. Al-Hindi R. R. Esmael A. Alotibi I. A. Azhari S. A. Alseghayer M. S. . (2022). The “big six”: hidden emerging foodborne bacterial pathogens. Trop. Med. Infect. Dis. 7, 356. doi: 10.3390/tropicalmed7110356, PMID: 36355898 PMC9693546

[B2] Anders S. Huber W. (2010). Differential expression analysis for sequence count data. Nat. Precedings, 11, R106. doi: 10.1186/gb-2010-11-10-r106, PMID: 20979621 PMC3218662

[B3] Baker R. G. Hayden M. S. Ghosh S. (2011). Nf-*κ*b, inflammation, and metabolic disease. Cell Metab. 13, 11–22. doi: 10.1016/j.cmet.2010.12.008, PMID: 21195345 PMC3040418

[B4] Batsaikhan B. Lin P.-C. Shigemura K. Wu Y.-W. Onishi R. Chang P.-R. . (2025). Comparison of global transcriptomes for nontyphoidal salmonella clinical isolates from pediatric patients with and without bacteremia after their interaction with human intestinal epithelial cells in *vitro*. J. Microbiology Immunol. Infection 58, 38–47. doi: 10.1016/j.jmii.2024.09.002, PMID: 39322508

[B5] Cardoso A. P. F. Banerjee M. Nail A. N. Lykoudi A. States J. C. (2021). mirna dysregulation is an emerging modulator of genomic instability. Semin. Cancer Biol. 76, 120–131. doi: 10.1016/j.semcancer.2021.05.004, PMID: 33979676 PMC8576067

[B6] Chen K.-H. Pan J.-F. Chen Z.-X. Pan D. Gao T. Huang M. . (2020). Effects of hsa circ 0000711 expression level on proliferation and apoptosis of hepatoma cells. Eur. Rev. Med. Pharmacol. Sci. 24, 4161–4171. doi: 10.26355/eurrev_202004_20996, PMID: 32373952

[B7] Detzner J. Pohlentz G. Müthing J. (2022). Enterohemorrhagic escherichia coli and a fresh view on shiga toxin-binding glycosphingolipids of primary human kidney and colon epithelial cells and their toxin susceptibility. Int. J. Mol. Sci. 23, 6884. doi: 10.3390/ijms23136884, PMID: 35805890 PMC9266556

[B8] Dias D. Costa S. Fonseca C. Baraúna R. Caetano T. Mendo S. (2022). Pathogenicity of shiga toxin-producing escherichia coli (stec) from wildlife: Should we care? Sci. Total Environ. 812, 152324. doi: 10.1016/j.scitotenv.2021.152324, PMID: 34915011

[B9] Fan W. Shi R. Guan M. Chen P. Wu H. Su W. . (2021). The effects of naringenin on mirna-mrna profiles in heparg cells. Int. J. Mol. Sci. 22, 2292. doi: 10.3390/ijms22052292, PMID: 33669020 PMC7956767

[B10] Gao X. Zhao D. Han J. Zhang Z. Wang Z. (2022). Identification of microrna–mrna–tf regulatory networks in periodontitis by bioinformatics analysis. BMC Oral. Health 22, 118. doi: 10.1186/s12903-022-02150-0, PMID: 35397550 PMC8994180

[B11] Géczi D. Klekner Á. Balogh I. Penyige A. Szilágyi M. Virga J. . (2025). Identification of deregulated mirnas and mrnas involved in tumorigenesis and detection of glioblastoma patients applying next-generation rna sequencing. Pharmaceuticals 18, 431. doi: 10.3390/ph18030431, PMID: 40143207 PMC11944724

[B12] Gelalcha B. D. Brown S. M. Crocker H. E. Agga G. E. Kerro Dego O. (2022). Regulation mechanisms of virulence genes in enterohemorrhagic escherichia coli. Foodborne Pathog. Dis. 19, 598–612. doi: 10.1089/fpd.2021.0103, PMID: 35921067

[B13] Gonzalez G. A. Cerqueira M. A. (2020). Shiga toxin-producing escherichia coli in the animal reservoir and food in Brazil. J. Appl. Microbiol. 128, 1568–1582. doi: 10.1111/jam.14500, PMID: 31650661

[B14] FAO/WHO STEC Expert Group . (2019). Hazard identification and characterization: Criteria for categorizing shiga toxin–producing escherichia coli on a risk basis. J. Food Prot. 82, 7–21. doi: 10.4315/0362-028X.JFP-18-291, PMID: 30586326

[B15] Han H. Cho J.-W. Lee S. Yun A. Kim H. Bae D. . (2018). Trrust v2: an expanded reference database of human and mouse transcriptional regulatory interactions. Nucleic Acids Res. 46, D380–D386. doi: 10.1093/nar/gkx1013, PMID: 29087512 PMC5753191

[B16] Huang D. W. Sherman B. T. Lempicki R. A. (2009). Systematic and integrative analysis of large gene lists using david bioinformatics resources. Nat. Protoc. 4, 44–57. doi: 10.1038/nprot.2008.211, PMID: 19131956

[B17] Huang P.-Y. Wu J.-G. Gu J. Zhang T.-Q. Li L.-F. Wang S.-Q. . (2021). Bioinformatics analysis of mirna and mrna expression profiles to reveal the key mirnas and genes in osteoarthritis. J. Orthopaedic Surg. Res. 16, 63. doi: 10.1186/s13018-021-02201-2, PMID: 33468167 PMC7814623

[B18] Kennedy J. Simmonds L. Orme R. Doherty W. (2017). An unusual case of escherichia coli o157: H7 infection with pseudomembranous colitis-like lesions associated with haemolytic-uraemic syndrome and neurological sequelae. Case Rep. 2017, bcr–2016. 10.1136/bcr-2016-218586 PMC5534687 28630239

[B19] Khamaneh A. M. Mohajeri N. Naghili B. Zarghami N. (2025). Profiling mrna and mirna expression variations associated with cyclin-dependent kinase pathway in the low-grade luminal early breast cancer. J. Appl. Genet. 66, 601–610. doi: 10.1007/s13353-024-00909-5, PMID: 39373948

[B20] Li X. Sun L. Chen L. Xu Y. Kong X. (2020). Upregulation of microrna-219-5p relieves ulcerative colitis through balancing the differentiation of treg/th17 cells. Eur. J. Gastroenterol. Hepatol. 32, 813–820. doi: 10.1097/MEG.0000000000001712, PMID: 32175983 PMC7269018

[B21] Liang Y. Zhou Y. Shen P. (2004). Nf-kappab and its regulation on the immune system. Cell Mol. Immunol. 1, 343–350. 16285893

[B22] Liu Y. Thaker H. Wang C. Xu Z. Dong M. (2022). Diagnosis and treatment for shiga toxin-producing escherichia coli associated hemolytic uremic syndrome. Toxins 15, 10. doi: 10.3390/toxins15010010, PMID: 36668830 PMC9862836

[B23] Liu X. Xiao H. Peng X. Chai Y. Wang S. Wen G. (2023). Identification and comprehensive analysis of circrna–mirna–mrna regulatory networks in osteoarthritis. Front. Immunol. 13, 1050743. doi: 10.3389/fimmu.2022.1050743, PMID: 36700234 PMC9869167

[B24] Mathusa E. C. Chen Y. Enache E. Hontz L. (2010). Non-o157 shiga toxin-producing Escherichia coli in foods. J. Food Prot. 73, 1721–1736. doi: 10.4315/0362-028X-73.9.1721, PMID: 20828483

[B25] Onyeka L. O. Adesiyun A. A. Keddy K. H. Madoroba E. Manqele A. Thompson P. N. (2020). Shiga toxin–producing escherichia coli contamination of raw beef and beef-based ready-to-eat products at retail outlets in pretoria, South Africa. J. Food Prot. 83, 476–484. doi: 10.4315/0362-028X.JFP-19-372, PMID: 32065651

[B26] Plotnikov A. Zehorai E. Procaccia S. Seger R. (2011). The mapk cascades: signaling components, nuclear roles and mechanisms of nuclear translocation. Biochim. Biophys. Acta (BBA)-Molecular Cell Res. 1813, 1619–1633. doi: 10.1016/j.bbamcr.2010.12.012, PMID: 21167873

[B27] Qin D. Zheng Y. Wang L. Lin Z. Yao Y. Fei W. . (2025). Unraveling shared diagnostic genes and cellular microenvironmental changes in endometriosis and recurrent implantation failure through multi-omics analysis. Sci. Rep. 15, 9110. doi: 10.1038/s41598-025-93146-7, PMID: 40097519 PMC11914081

[B28] Scallan E. Hoekstra R. M. Angulo F. J. Tauxe R. V. Widdowson M.-A. Roy S. L. . (2011). Foodborne illness acquired in the United States—major pathogens. Emerging Infect. Dis. 17, 7. doi: 10.3201/eid1701.P11101, PMID: 21192848 PMC3375761

[B29] Song Z. Feng Z. Wang X. Li J. Zhang D. (2025). Nfkb1 as a key player in tumor biology: from mechanisms to therapeutic implications. Cell Biol. Toxicol. 41, 29. doi: 10.1007/s10565-024-09974-2, PMID: 39797972 PMC11724797

[B30] Song D. Lee J. Kwak W. Song M. Oh H. Kim Y. . (2022). Stimbiotic supplementation alleviates poor performance and gut integrity in weaned piglets induced by challenge with e. coli. Animals 12, 1799. doi: 10.3390/ani12141799, PMID: 35883346 PMC9312148

[B31] Szklarczyk D. Gable A. L. Lyon D. Junge A. Wyder S. Huerta-Cepas J. . (2019). String v11: protein–protein association networks with increased coverage, supporting functional discovery in genome-wide experimental datasets. Nucleic Acids Res. 47, D607–D613. doi: 10.1093/nar/gky1131, PMID: 30476243 PMC6323986

[B32] Wang M.-Y. Liu W.-J. Wu L.-Y. Wang G. Zhang C.-L. Liu J. (2023). The research progress in transforming growth factor-*β*2. Cells 12, 2739. doi: 10.3390/cells12232739, PMID: 38067167 PMC10706148

[B33] Wang W. Wang H. Liu Y. Yang L. (2022). Identification of mirna-mrna-tf regulatory networks in peripheral blood mononuclear cells of type 1 diabetes. BMC Endocrine Disord. 22, 119. doi: 10.1186/s12902-022-01038-y, PMID: 35534828 PMC9087960

[B34] Watson G. Ze’ev A. R. Lau E. (2017). Atf2, a paradigm of the multifaceted regulation of transcription factors in biology and disease. Pharmacol. Res. 119, 347–357. doi: 10.1016/j.phrs.2017.02.004, PMID: 28212892 PMC5457671

[B35] Wu Z.-H. Wang Y.-X. Song J.-J. Zhao L.-Q. Zhai Y.-J. Liu Y.-F. . (2024). Lncrna snhg26 promotes gastric cancer progression and metastasis by inducing c-myc protein translation and an energy metabolism positive feedback loop. Cell Death Dis. 15, 236. doi: 10.1038/s41419-024-06607-8, PMID: 38553452 PMC10980773

[B36] Xu M. Zhou J. Zhang Q. Le K. Xi Z. Yi P. . (2020). Mir-3121-3p promotes tumor invasion and metastasis by suppressing rap1gap in papillary thyroid cancer in *vitro*. Ann. Trans. Med. 8, 1229. doi: 10.21037/atm-20-4469, PMID: 33178761 PMC7607113

[B37] Yang J. Song H. Cao K. Song J. Zhou J. (2019). Comprehensive analysis of helicobacter pylori infection-associated diseases based on mirna-mrna interaction network. Briefings Bioinf. 20, 1492–1501. doi: 10.1093/bib/bby018, PMID: 29579224 PMC6781589

[B38] Zhang Q. Liu W. Zhang H.-M. Xie G.-Y. Miao Y.-R. Xia M. . (2020). htftarget: a comprehensive database for regulations of human transcription factors and their targets. Genomics Proteomics Bioinf. 18, 120–128. doi: 10.1016/j.gpb.2019.09.006, PMID: 32858223 PMC7647694

[B39] Zhang L. Ma X. Tong P. Zheng B. Zhu M. Peng B. . (2024). Rna-seq analysis of long non-coding rna in human intestinal epithelial cells infected by shiga toxin-producing escherichia coli. Cytokine 173, 156421. doi: 10.1016/j.cyto.2023.156421, PMID: 37944420

[B40] Zhang X. McDaniel A. D. Wolf L. E. Keusch G. T. Waldor M. K. Acheson D. W. (2000). Quinolone antibiotics induce shiga toxin-encoding bacteriophages, toxin production, and death in mice. J. Infect. Dis. 181, 664–670. doi: 10.1086/315239, PMID: 10669353

[B41] Zhang W. Zhang Q. Che L. Xie Z. Cai X. Gong L. . (2022). Using biological information to analyze potential mirna-mrna regulatory networks in the plasma of patients with non-small cell lung cancer. BMC Cancer 22, 299. doi: 10.1186/s12885-022-09281-1, PMID: 35313857 PMC8939143

[B42] Zhao T. Gao P. Li Y. Tian H. Ma D. Sun N. . (2023). Investigating the role of fads family members in breast cancer based on bioinformatic analysis and experimental validation. Front. Immunol. 14, 1074242. doi: 10.3389/fimmu.2023.1074242, PMID: 37122728 PMC10130515

[B43] Zheng H. Liu M. Shi S. Huang H. Yang X. Luo Z. . (2024). Map4k4 and wt1 mediate sox6- induced cellular senescence by synergistically activating the atf2–tgf*β*2–smad2/3 signaling pathway in cervical cancer. Mol. Oncol. 18, 1327–1346. doi: 10.1002/1878-0261.13613, PMID: 38383842 PMC11076992

[B44] Zhou R. Chen Z. Hao D. Wang Y. Zhang Y. Yi X. . (2019). Enterohemorrhagic escherichia coli tir inhibits tak1 activation and mediates immune evasion. Emerging Microbes Infections 8, 734–748. doi: 10.1080/22221751.2019.1620589, PMID: 31130074 PMC6542180

