# Supplementary material for: Integrated transcriptomic analysis reveals miRNA-hub mRNA-TF interactions and key regulatory targets in STEC infected intestinal epithelial cells
Source: Front Cell Infect Microbiol. 2026 Apr 2;16:1772607. doi: 10.3389/fcimb.2026.1772607 (PMC13083180; doi:10.3389/fcimb.2026.1772607)
Supplement: Supplementary file 2 [file Table1.docx]

Table S1 Reverse transcription primers and qPCR primers

| **Primer name** | **Sequences (5’-3’)** |
| --- | --- |
| miR-33-RT | GTCGTATCCAGTGCAGGGTCCGAGGTATTCGCACTGGATACGACGGGCTG |
| miR-370-RT | GTCGTATCCAGTGCAGGGTCCGAGGTATTCGCACTGGATACGACACCAGG |
| miR-543-RT | GTCGTATCCAGTGCAGGGTCCGAGGTATTCGCACTGGATACGACAAGAAG |
| miR-3065-RT | GTCGTATCCAGTGCAGGGTCCGAGGTATTCGCACTGGATACGACCTCCAA |
| U6-RT | GTCGTATCCAGTGCAGGGTCCGAGGTATTCGCACTGGATACGACAAAATA |
| qPCR-miR-R  (common use) | ATCCAGTGCAGGGTCCGAGG |
| miR-33-F | AAGTATTCCAGTGCCTCGGCAG |
| miR-370-F | AACTTGATGCCTGCTGGGGTG |
| miR-543-F | ACGGCACAAACATTCGCGGT |
| miR-3065-F | AAGCTGAGTCAGCACCAGGATATT |
| U6-F | CTCGCTTCGGCAGCACA |
| PIK3CA-F | CTGTCTCCTCTAAACCCTGCTC |
| PIK3CA-R | TATCTTGCCGTAAATCATCCC |
| IL6-F | CTTCGGTCCAGTTGCCTTCT |
| IL6-R | GCCTCTTTGCTGCTTTCACA |
| BBC3-F | GACCTCAACGCACAGTACGAG |
| BBC3-R | AGGAGTCCCATGATGAGATTGT |
| FOSL1-F | TACCTTGTATCTCCCTTTCCC |
| FOSL1-R | TGCTGCTACTCTTGCGATGA |
| GAPDH-F | ACAACTTTGGTATCGTGGAAGG |
| GAPDH-R | GCCATCACGCCACAGTTTC |
