# Supplementary material for: Integrated transcriptomic analysis reveals miRNA-hub mRNA-TF interactions and key regulatory targets in STEC infected intestinal epithelial cells
Source: Front Cell Infect Microbiol. 2026 Apr 2;16:1772607. doi: 10.3389/fcimb.2026.1772607 (PMC13083180; doi:10.3389/fcimb.2026.1772607)
Supplement: Supplementary file 3 [file Table2.docx]

**Table S2** TRRUST based prediction of transcription factors regulating the 10 hub genes

| **Key TF** | **Description** | **overlapped genes** |
| --- | --- | --- |
| NR4A1 | nuclear receptor subfamily 4, group A, member 1 | 2 |
| E2F1 | E2F transcription factor 1 | 3 |
| ERG | v-ets erythroblastosis virus E26 oncogene homolog (avian) | 2 |
| HSF1 | heat shock transcription factor 1 | 2 |
| ATF2 | activating transcription factor 2 | 2 |
| USF2 | upstream transcription factor 2, c-fos interacting | 2 |
| RELA | v-rel reticuloendotheliosis viral oncogene homolog A (avian) | 3 |
| NFKB1 | nuclear factor of kappa light polypeptide gene enhancer in B-cells 1 | 3 |
| CEBPB | CCAAT/enhancer binding protein (C/EBP), beta | 2 |
| USF1 | upstream transcription factor 1 | 2 |
| HIF1A | hypoxia inducible factor 1, alpha subunit (basic helix-loop-helix transcription factor) | 2 |
| EGR1 | early growth response 1 | 2 |
| JUN | jun proto-oncogene | 2 |
| SP1 | Sp1 transcription factor | 2 |
