# Supplementary material for: Integrated transcriptomic analysis reveals miRNA-hub mRNA-TF interactions and key regulatory targets in STEC infected intestinal epithelial cells
Source: Front Cell Infect Microbiol. 2026 Apr 2;16:1772607. doi: 10.3389/fcimb.2026.1772607 (PMC13083180; doi:10.3389/fcimb.2026.1772607)
Supplement: Supplementary file 4 [file Table3.doc]

**Table S3** TFtarget based prediction of transcription factors regulating the 10 hub genes

| **TF** | **Full name** | **overlapped genes** |
| --- | --- | --- |
| SMC1A | structural maintenance of chromosomes 1A | 10 |
| TP53 | tumor protein p53 | 10 |
| RAD21 | RAD21 cohesin complex component | 10 |
| ERG | ERG, ETS transcription factor | 10 |
| NFKB1 | nuclear factor kappa B subunit 1 | 10 |
| FLI1 | Fli-1 proto-oncogene, ETS transcription factor | 9 |
| ETS1 | ETS proto-oncogene 1, transcription factor | 9 |
| TEAD4 | TEA domain transcription factor 4 | 9 |
| EHMT2 | euchromatic histone lysine methyltransferase 2 | 9 |
| JUN | Jun proto-oncogene, AP-1 transcription factor subunit | 9 |
| ATF3 | activating transcription factor 3 | 9 |
| ZNF382 | zinc finger protein 382 | 9 |
| EP300 | E1A binding protein p300 | 9 |
| NEUROG2 | neurogenin 2 | 9 |
| BCOR | BCL6 corepressor | 8 |
| NR2F2 | nuclear receptor subfamily 2 group F member 2 | 8 |
| MAZ | MYC associated zinc finger protein | 8 |
| FOXO1 | forkhead box O1 | 8 |
| YAP1 | Yes associated protein 1 | 8 |
| EGR2 | early growth response 2 | 8 |
| PPARG | peroxisome proliferator activated receptor gamma | 8 |
| HEXIM1 | hexamethylene bisacetamide inducible 1 | 8 |
| KDM2B | lysine demethylase 2B | 8 |
| PCGF1 | polycomb group ring finger 1 | 8 |
| HDAC2 | histone deacetylase 2 | 8 |
| CTCF | CCCTC-binding factor | 8 |
| EGR3 | early growth response 3 | 8 |
| GLIS1 | GLIS family zinc finger 1 | 8 |
| GRHL3 | grainyhead like transcription factor 3 | 8 |
| TAZ | tafazzin | 8 |
| FOXA1 | forkhead box A1 | 8 |
| NR1H3 | nuclear receptor subfamily 1 group H member 3 | 8 |
| FOXA2 | forkhead box A2 | 8 |
| BRD4 | bromodomain containing 4 | 7 |
| FOSL1 | FOS like 1, AP-1 transcription factor subunit | 7 |
| NRF1 | nuclear respiratory factor 1 | 7 |
| ZNF35 | zinc finger protein 35 | 7 |
| CREB1 | cAMP responsive element binding protein 1 | 7 |
| AZF1 | azoospermia factor 1 | 7 |
| GABPA | GA binding protein transcription factor alpha subunit | 7 |
| ZSCAN22 | zinc finger and SCAN domain containing 22 | 7 |
| TET2 | tet methylcytosine dioxygenase 2 | 7 |
| ZNF341 | zinc finger protein 341 | 7 |
| JUNB | JunB proto-oncogene, AP-1 transcription factor subunit | 7 |
| CEBPB | CCAAT/enhancer binding protein beta | 7 |
| SP2 | Sp2 transcription factor | 7 |
| ATF1 | activating transcription factor 1 | 7 |
| ELF3 | E74 like ETS transcription factor 3 | 7 |
| SKI | SKI proto-oncogene | 7 |
| FOXP1 | forkhead box P1 | 7 |
| PGR | progesterone receptor | 7 |
| SP1 | Sp1 transcription factor | 7 |
| RYBP | RING1 and YY1 binding protein | 7 |
| HDAC1 | histone deacetylase 1 | 6 |
| CDK9 | cyclin dependent kinase 9 | 6 |
| TCF3 | transcription factor 3 | 6 |
| SPI1 | Spi-1 proto-oncogene | 6 |
| IRF1 | interferon regulatory factor 1 | 6 |
| ZNF263 | zinc finger protein 263 | 6 |
| LARP7 | La ribonucleoprotein domain family member 7 | 6 |
| RELA | RELA proto-oncogene, NF-kB subunit | 6 |
| RUNX1 | runt related transcription factor 1 | 6 |
| KLF4 | Kruppel like factor 4 | 6 |
| ZBTB48 | zinc finger and BTB domain containing 48 | 6 |
| TRIM28 | tripartite motif containing 28 | 6 |
| ZBTB2 | zinc finger and BTB domain containing 2 | 6 |
| CEBPA | CCAAT/enhancer binding protein alpha | 6 |
| GRHL2 | grainyhead like transcription factor 2 | 6 |
| BPTF | bromodomain PHD finger transcription factor | 6 |
| NFE2 | nuclear factor, erythroid 2 | 6 |
| USF1 | upstream transcription factor 1 | 6 |
| SP4 | Sp4 transcription factor | 6 |
| NKX2-1 | NK2 homeobox 1 | 6 |
| GTF2B | general transcription factor IIB | 6 |
| MYF5 | myogenic factor 5 | 6 |
| ZBTB14 | zinc finger and BTB domain containing 14 | 6 |
| SIX2 | SIX homeobox 2 | 6 |
| CEBPD | CCAAT/enhancer binding protein delta | 6 |
| SCRT1 | scratch family transcriptional repressor 1 | 6 |
| ZFP64 | ZFP64 zinc finger protein | 6 |
| ZNF384 | zinc finger protein 384 | 5 |
| SUMO2 | small ubiquitin-like modifier 2 | 5 |
| LMNB1 | lamin B1 | 5 |
| PLAG1 | PLAG1 zinc finger | 5 |
| JMJD1C | jumonji domain containing 1C | 5 |
| GATA1 | GATA binding protein 1 | 5 |
| NR4A1 | nuclear receptor subfamily 4 group A member 1 | 5 |
| STAT3 | signal transducer and activator of transcription 3 | 5 |
| MED1 | mediator complex subunit 1 | 5 |
| FEZF1 | FEZ family zinc finger 1 | 5 |
| KLF5 | Kruppel like factor 5 | 5 |
| NFIC | nuclear factor I C | 5 |
| MED12 | mediator complex subunit 12 | 5 |
| TBX5 | T-box 5 | 5 |
| MYOD1 | myogenic differentiation 1 | 5 |
| EZH1 | enhancer of zeste 1 polycomb repressive complex 2 subunit | 5 |
| STAT1 | signal transducer and activator of transcription 1 | 4 |
| KDM5B | lysine demethylase 5B | 4 |
| BRD2 | bromodomain containing 2 | 4 |
| E2F6 | E2F transcription factor 6 | 4 |
| KLF9 | Kruppel like factor 9 | 4 |
| MYH11 | myosin heavy chain 11 | 4 |
| RNF2 | ring finger protein 2 | 4 |
| PBX1 | PBX homeobox 1 | 4 |
| NFKB2 | nuclear factor kappa B subunit 2 | 4 |
| ZNF574 | zinc finger protein 574 | 4 |
| TAL1 | TAL bHLH transcription factor 1, erythroid differentiation factor | 4 |
| SOX4 | SRY-box 4 | 4 |
| ILF3 | interleukin enhancer binding factor 3 | 4 |
| ZSCAN5A | zinc finger and SCAN domain containing 5A | 4 |
| ZNF770 | zinc finger protein 770 | 4 |
| APC | APC, WNT signaling pathway regulator | 4 |
| SPDEF | SAM pointed domain containing ETS transcription factor | 4 |
| NR1H2 | nuclear receptor subfamily 1 group H member 2 | 4 |
| NFIA | nuclear factor I A | 4 |
| HOXB13 | homeobox B13 | 4 |
| NANOG | Nanog homeobox | 4 |
| TWIST1 | twist family bHLH transcription factor 1 | 4 |
| E2F1 | E2F transcription factor 1 | 4 |
| MAX | MYC associated factor X | 4 |
| KLF15 | Kruppel like factor 15 | 4 |
| TFAP2C | transcription factor AP-2 gamma | 4 |
| ZNF467 | zinc finger protein 467 | 4 |
| ZBTB26 | zinc finger and BTB domain containing 26 | 4 |
| OTX2 | orthodenticle homeobox 2 | 4 |
| NOTCH1 | notch 1 | 3 |
| RUNX3 | runt related transcription factor 3 | 3 |
| WDR5 | WD repeat domain 5 | 3 |
| TFAP4 | transcription factor AP-4 | 3 |
| STAG1 | stromal antigen 1 | 3 |
| ZBTB7A | zinc finger and BTB domain containing 7A | 3 |
| RBBP5 | RB binding protein 5, histone lysine methyltransferase complex subunit | 3 |
| RELB | RELB proto-oncogene, NF-kB subunit | 3 |
| MYB | MYB proto-oncogene, transcription factor | 3 |
| LYL1 | LYL1, basic helix-loop-helix family member | 3 |
| EBF3 | early B-cell factor 3 | 3 |
| MECOM | MDS1 and EVI1 complex locus | 3 |
| GATA3 | GATA binding protein 3 | 3 |
| ZNF121 | zinc finger protein 121 | 3 |
| TBX21 | T-box 21 | 3 |
| CREBBP | CREB binding protein | 3 |
| TCF4 | transcription factor 4 | 3 |
| MAF | MAF bZIP transcription factor | 3 |
| USF2 | upstream transcription factor 2, c-fos interacting | 3 |
| ZNF554 | zinc finger protein 554 | 3 |
| PIAS1 | protein inhibitor of activated STAT 1 | 3 |
| NR3C1 | nuclear receptor subfamily 3 group C member 1 | 3 |
| SMARCC1 | SWI/SNF related, matrix associated, actin dependent regulator of chromatin subfamily c member 1 | 3 |
| AR | androgen receptor | 3 |
| PBX3 | PBX homeobox 3 | 3 |
| EWSR1 | EWS RNA binding protein 1 | 3 |
| ARID3A | AT-rich interaction domain 3A | 3 |
| ZBTB42 | zinc finger and BTB domain containing 42 | 3 |
| KLF10 | Kruppel like factor 10 | 3 |
| ZNF528 | zinc finger protein 528 | 3 |
| ARNT | aryl hydrocarbon receptor nuclear translocator | 3 |
| SMC3 | structural maintenance of chromosomes 3 | 3 |
| ZEB1 | zinc finger E-box binding homeobox 1 | 3 |
| FOSL2 | FOS like 2, AP-1 transcription factor subunit | 3 |
| ZSCAN5DP | zinc finger and SCAN domain containing 5D pseudogene | 3 |
| EPAS1 | endothelial PAS domain protein 1 | 3 |
| ESR1 | estrogen receptor 1 | 3 |
| ASXL1 | additional sex combs like 1, transcriptional regulator | 3 |
| DDX5 | DEAD-box helicase 5 | 2 |
| EBF1 | early B-cell factor 1 | 2 |
| CBFB | core-binding factor beta subunit | 2 |
| STAT5B | signal transducer and activator of transcription 5B | 2 |
| IRF4 | interferon regulatory factor 4 | 2 |
| PHF8 | PHD finger protein 8 | 2 |
| REL | REL proto-oncogene, NF-kB subunit | 2 |
| SUPT5H | SPT5 homolog, DSIF elongation factor subunit | 2 |
| ZNF18 | zinc finger protein 18 | 2 |
| BATF | basic leucine zipper ATF-like transcription factor | 2 |
| CDK12 | cyclin dependent kinase 12 | 2 |
| ZNF639 | zinc finger protein 639 | 2 |
| TLX1 | T-cell leukemia homeobox 1 | 2 |
| DRAP1 | DR1 associated protein 1 | 2 |
| ZSCAN16 | zinc finger and SCAN domain containing 16 | 2 |
| JUND | JunD proto-oncogene, AP-1 transcription factor subunit | 2 |
| OSR2 | odd-skipped related transciption factor 2 | 2 |
| SRF | serum response factor | 2 |
| MYBL2 | MYB proto-oncogene like 2 | 2 |
| ATF2 | activating transcription factor 2 | 2 |
| TCF21 | transcription factor 21 | 2 |
| HES1 | hes family bHLH transcription factor 1 | 2 |
| ARID1B | AT-rich interaction domain 1B | 2 |
| TSC22D4 | TSC22 domain family member 4 | 2 |
| ARID1A | AT-rich interaction domain 1A | 2 |
| SMARCB1 | SWI/SNF related, matrix associated, actin dependent regulator of chromatin, subfamily b, member 1 | 2 |
| ARID2 | AT-rich interaction domain 2 | 2 |
| FOXO3 | forkhead box O3 | 2 |
| PRDM1 | PR/SET domain 1 | 2 |
| ETV1 | ETS variant 1 | 2 |
| NRIP1 | nuclear receptor interacting protein 1 | 2 |
| ZBTB17 | zinc finger and BTB domain containing 17 | 2 |
| HCFC1 | host cell factor C1 | 2 |
| CBX8 | chromobox 8 | 2 |
| KLF1 | Kruppel like factor 1 | 2 |
| SIX5 | SIX homeobox 5 | 2 |
| MYCN | MYCN proto-oncogene, bHLH transcription factor | 2 |
| REST | RE1 silencing transcription factor | 2 |
| CRY1 | cryptochrome circadian regulator 1 | 2 |
| KLF6 | Kruppel like factor 6 | 2 |
| SMAD4 | SMAD family member 4 | 2 |
| HNF1B | HNF1 homeobox B | 2 |
| ASCL1 | achaete-scute family bHLH transcription factor 1 | 2 |
| MYOG | myogenin | 2 |
| PRDM6 | PR/SET domain 6 | 2 |
